# Dropsy—Complicated with Aneurism

**Published:** 1884-12

**Authors:** J. McF. Gaston

**Affiliations:** Professor of Principles and Practice of Surgery


### DROPSY COMPLICATED WITH ANEURISM.

A CLINICAL LECTURE DELIVERED ON THE 10TH OF OCTOBER, 1884, IN THE SOUTHERN MEDICAL COLLEGE, ATLANTA, GA.

---

BY DR. J. McF. GASTON, PROFESSOR OF PRINCIPLES AND PRACTICE OF SURGERY.

---

The case presented to you affords interesting points of study by the immense accumulation of fluid in the abdominal cavity, and the distension of the lower limbs, connected probably with an ovarian tumor on the right side, while there is, as you see, a pulsating tumor at the base of the neck, immediately above the inner portion of the right clavicle. Each of you may profit by examining the fluctuation in the peritoneal sac, and the bruit in the aneurismal tumor, while the preparations are going on for the operation of tapping, known technically as peracentesis abdominis. You observe that I have a broad bandage, divided at each extremity into four strips, which being passed around the upper part of the abdominal protuberance, the ends are interlaced at the back of the patient, while one of your companions on either side of the table secures all four together, so as to make steady traction while the fluid is discharged. It will be noted that there are two superficial ulcers on the anterior aspect of the left leg, and an irregular extension of vesicles upon the right, resulting from the yielding of the tissues under the pressure of the serous accumulation in these parts, and that the whole extent of the lower extremities has become swollen from distention with fluid. This black woman reports herself to be fifty years old, and to have borne eleven children, with one abortion some six years ago, with disappearance of the menstrual flow two years subsequently. Her troubles commenced some three years since, but there was no marked local prominence observed on either side of the abdomen previous to the general distention within the past year, and she was not conscious of the pulsating tumor at the lower anterior part of the neck until within a few months past, which, as you perceive, bulges up distinctly about the size of a pullet's egg, above the line of the clavicle. It is observed also that the external jugular vein upon the same side is considerably enlarged, owing doubtless to more or less obstruction by pressure of the tumor; and deepseated compression undoubtedly is made upon the descending vena cava, while the ascending vena cava must sustain some impediment to the free flow of blood by the great distention of the abdominal cavity with the serous fluid. This dropsical effusion is simply an exudation of the watery portion of the blood, and is a consequence of impairment in the venous circulation, which induces the passage of this serous fluid into the cellular tissue under the skin, that constitutes the diffuse swelling known as anasarca, or into the cavity of the peritoneum, within the abdomen, known as ascites, both of which conditions are strikingly shown in the patient before us. I may state that visceral obstruction is a frequent source of this impairment in the absorptive processes, that leads to dropsical effusion; but there are two distinct organs which by their derangements induce serous exudations, and it is to the liver and the heart, that most cases of dropsy are to be traced. Those having their origin in hepatic disorders are generally amenable to treatment, while those originating from cardiac troubles prove for the most part intractable. The auscultation over the precordial region in this case leads to the inference of cardiac insufficiency, and the pulsation is imperceptible not only in the radial artery, but along the course of the brachial, and in the axillary it is very indistinct, so that we may attribute the effusion from the vascular tissues to the lack of energy in the central organ of the circulation, and make our prognosis accordingly.

Preparatory to bringing this case before you, she was given for two days in succession a combination of pulverized squill 3 grains, and calomel 1 grain, every three hours; and this morning early she took twenty (20) grains of jalap, with half an ounce of cream of tartar, which has already operated freely as a cathartic. After drawing off the water she will take a dose of the squill and calomel three times a day, by which the general serous exudation in the cellular tissues may be entirely removed for a time, and the ulcers on her legs will most likely heal under the constant application of the roller bandage, to give a gentle support to the cutaneous surfaces. But these improvements can only be permanent by relieving the cardiac insufficiency, which is the prime cause of trouble.

Having all things now in readiness, as you observe, for proceeding with the requisite operation, it may not be out of place to draw your attention to the fact that the thickening of the tissues in the linea alba, beneath the umbilicus, from the anasarca, renders it inexpedient to make the puncture, as I usually do, at a point in this line one-third distant from the umbilicus to the pubic bone. Finding on the right iliac region, that the sense of fluctuation is less distinct than on the left side, I will make the puncture at a point midway between the umbilicus and the left superior spinous process of the ilium. After passing glycerine over the trocar, which is held securely in my right hand, the thumb and finger of my left hand are placed on either side of the selected spot, not simply to give tension to the skin, but to be in position to seize the sheath, when the trocar point is withdrawn. It should be noted that a sudden thrust of the instrument to the depth required is made, and the fluid is coming freely from the canula that penetrates within the cavity of the abdomen.

You are amused at the patient's apprehension that I have punctured her bladder, and she labors perhaps under the delusion that the urine has been all this time accumulating within the body. While in this instance there is no doubt on this point, it is advisable when the puncture is made in the linea alba to use the catheter before using the trocar, and this even when the declaration is made that the urine has been regularly discharged, as there are cases in which enormous distention of the bladder has ensued from the overplus of urine after micturition daily.

It might be supposed that there was danger to the intestines in thrusting a trocar some two and a half inches into the peritoneal cavity, as I have done here, but apart from the bowels being bound to the posterior wall of the abdomen by the mesentery, the coils of the intestine float so loosely in the fluid that they would recede from the point of the instrument without being wounded. While one of your companions is emptying the small basin as it fills, into a larger reservoir for the dropsical discharges, the other two are gradually tightening the broad bandage, so as to keep up a gradual support in place of the distention, and thus relieve the sense of sinking and discomfort which would otherwise follow the removal of the fluid from the cavity. Now that a considerable reduction in the size of the abdomen has been effected, I can better discern on the right side extending from the iliac into the hypochondriac region an indurated mass, whose outline cannot, however, be distinctly defined, from the gross anasarcous state of the parietes of the abdomen; and hence it is still questionable whether it be an ovarian tumor or the enlargement of some other structure. It is to be noted that the water ceases to flow while there is marked abdominal protuberance, and that there is a distinct tympanitic distention of the epigastric and hypochondriac spaces, indicating a considerable gaseous collection in the colon, that will most likely disappear with the compression of the bandage, which is now secured with simple compresses of old soft cloth over the aperture left upon removal of the sheath of the trocar. This was accomplished, as you observed, by pressing the thumb and forefinger against the skin while the canula was drawn out from the tight grasp of the contracted tissues. By the withdrawal of such a quantity of water, which may be weighed or measured for more accurate information, the upward pressure upon the thoracic viscera has been removed, and, as you perceive, the aneurismal tumor is not so prominent, so that there may be a better prospect of a spontaneous cure, as no operation is practicable in this case. The disease is located doubtless in the arch of the aorta, and only general measures, calculated to lessen the force of the circulation, or lessen the impediments to the flow of the blood, can be used. Strange as it may seem, relief sometimes comes in these dilatations of the arterial coats from depositions of layers of fibrinous matter within their sac, it being thus altogether obliterated and the canal becoming reduced to its natural caliber. I have seen an interthoracic aneurism work its way through the upper portion of the breastbone, and still undergo spontaneous obliteration, so that the operation of nature avails at times when there is no opportunity for resorting to the resources of art. This patient will be kept under observation.

The most general division of aneurism is into the internal and external, this case being one of the former, while those located in the outer parts of the arterial system belong to the latter. They are also classified as true and false; this being a specimen of true aneurism in which the coats of the artery are dilated ; and the false consisting in escape of blood into the surrounding tissues from a lesion of the vessel. Another variety, known as varicose aneurism, results from the communication of the artery with a vein, as occurs sometimes in bloodletting at the bend of the arm. Although the case before us does not call for any special surgical interference, it may be stated that the gradual dilatation in the fusiform and in the saculated aneurism of the external order leaves the elastic, the muscular and fibrous coats so attenuated, or in part obliterated, that rupture is liable to ensue, and hence it is requisite to obviate a serious result, either by pressure or by ligation of the vessel on the cardiac or distal side of the tumors. While others have reported satisfactory results from uninterrupted pressure in the line of the arterial track leading to the aneurism, it has not yielded good results in the occasional experiments I have made with it, and some instances have occurred in which a partial coagula has formed which temporarily arrested the flow of blood in the tumor, but subsequently it has given way, and the fragments have been urged forward so as to obstruct the small vessels by emboli or thrombosis. We have had in a Journal very recently a report of death from this cause, so that the treatment by pressure cannot be relied upon as efficient or safe. The radical cure is by ligation, and I have repeatedly tied the femoral artery for aneurism in the popliteal region, without any untoward accident, and with the most satisfactory result in the complete obliteration of the tumor and restoration of the circulation by the anastimosing vessels. It is not requisite that the arterial trunk shall be ligated very near the tumor and soundness in the coats is better secured by ligation at a point somewhat remote from the dilatation.

The plan of masterly inactivity to be adopted in the present case may be watched by you with interest.

